# Development of a Mortality Prediction Model for Incarcerated Adults to Identify Palliative Care Needs

**DOI:** 10.1007/s11606-025-10103-w

**Published:** 2025-12-18

**Authors:** W. James Deardorff, Alexandra K. Lee, Kaiwei Lu, Bocheng Jing, W. John Boscardin, Michele DiTomas, John Dunlap, Brie A. Williams, Sei J. Lee, Alexander K. Smith

**Affiliations:** 1 Division of Geriatrics, University of California, San Francisco, San Francisco, CA, USA; 2 Philip R. Lee Institute for Health Policy Studies, University of California San Francisco, San Francisco, CA, USA; 3 Northern California Institute for Research and Education, San Francisco, CA, USA; 4 Department of Epidemiology and Biostatistics, University of California, San Francisco, San Francisco, CA, USA; 5 California Department of Corrections and Rehabilitation, Elk Grove, CA, USA; 6 Division of Health and Society, University of California, San Francisco, San Francisco, CA, USA

**Keywords:** prognostic model, mortality, incarceration, compassionate release, palliative care

## Abstract

**BACKGROUND:**

Estimating mortality risk in incarcerated adults is important for identifying individuals who may benefit from palliative care and compassionate release referrals.

**OBJECTIVE:**

To develop and internally validate a 2-year mortality prediction model in incarcerated adults.

**DESIGN:**

Cohort study (February 1, 2018–February 1, 2020).

**PARTICIPANTS:**

Incarcerated adults aged ≥ 18 years residing at a California Department of Corrections and Rehabilitation (CDCR) prison for ≥ 1 year.

**MAIN MEASURES:**

Model predictors included demographics (e.g., age, sex), housing status (general housing vs. higher acuity infirmary bed vs. lower acuity infirmary bed), functional assessment (level of mobility restriction), healthcare utilization (e.g., hospitalizations and intensive care unit admissions in the previous year), and chronic conditions. The primary outcome was natural death at 2 years, defined as death due to causes other than suicide, homicide, accidental injury, or drug overdose. Cox proportional hazards regression with LASSO for variable selection was used to develop the model. Model performance was assessed by discrimination (area under the receiver operating characteristic curve (AUC) at 2 years) and calibration (plots of predicted and observed mortality). Classification metrics were assessed at clinically relevant thresholds.

**KEY RESULTS:**

The final cohort included 89,430 adults (median age 40 years (interquartile range = 20), 10.2% ≥ 60 years, 30.6% Black, 41.4% Hispanic). At 2 years, 506 (0.6%) individuals experienced a natural death. The optimism-corrected AUC at 2 years after bootstrap internal validation was 0.926 (95% confidence interval (CI) = 0.915–0.938). The calibration plot at 2 years suggested good calibration. At a 2-year mortality risk threshold of 5%, sensitivity, specificity, and positive predictive value were 47.6% (95% CI = 42.3–51.6%), 98.4% (95% CI = 98.2–98.4%), and 16.7% (95% CI = 14.3–18.5%), respectively.

**CONCLUSIONS:**

The mortality risk estimates from this model can help clinicians identify individuals who may most benefit from advance care planning discussions, palliative care services, and compassionate release referrals.

## INTRODUCTION

The United States prison population has seen a rapid rise in the number of older adults, with roughly 14% of male prisoners and 9% of female prisoners aged 55 years or older in 2020.^1,2^ Incarcerated adults experience accelerated aging, leading to reduced life expectancy and higher rates of chronic medical conditions, functional impairments, and mental health conditions compared with non-incarcerated persons.^3-5^ For individuals with advanced age, multimorbidity, and/or serious illness, advance care planning discussions, palliative care, and hospice services are crucial for improving quality of life and ensuring medical care that is consistent with an individual’s values and goals.^6^ As the number of incarcerated adults with limited life expectancy increases, there is a clear need for a systematic way to identify individuals who may most benefit from these services.

Palliative care and hospice programs within the prison system have grown over the last few decades.^7,8^ However, these programs are frequently underutilized within the prison system due to limited access to consultations (e.g., prolonged wait times, lack of specialists), uncertainty among healthcare professionals regarding protocols for providing palliative care, and delayed recognition of end of life trajectories by healthcare teams who predominantly care for incarcerated individuals without serious illness.^7-9^ Similarly, there are several unique barriers to advance care planning in the prison system, including provider uncertainty and legal concerns, restrictive institutional policies, patient isolation from family/friends, and patient mistrust of the correctional healthcare system.^10,11^

Additionally, most states within the United States have compassionate release programs that allow for early release of incarcerated individuals with, for example, a serious and advanced illness with an end-of-life trajectory (e.g., metastatic cancer, advanced dementia). However, these programs have historically been used infrequently.^7,12^ In one study of several states with compassionate release policies, 5,932 persons were eligible for release between 2013–2015, but fewer than half applied (46.4%; *n* = 2,751) and only 802 (29.2%) were ultimately discharged through compassionate release.^13^ Low utilization of compassionate release is multi-factorial, including narrow eligibility requirements, limited awareness, and lack of knowledge around end-of-life trajectories.^12^ Identifying individuals with palliative care needs and those who may be eligible for compassionate release is therefore essential to humanely caring for incarcerated individuals with serious illness.^14^

Prognostic models can assist in identifying individuals at high risk of mortality who may benefit from either advance care planning discussions, palliative care and hospice referrals, or compassionate release. Prognostic models have been widely used across a variety of settings, including community-dwelling older adults, individuals with cancer, and hospitalized individuals.^15^ However, there are currently no prognostic tools for incarcerated individuals, a uniquely vulnerable population with distinct health needs and circumstances. Therefore, in collaboration with the California Department of Corrections and Rehabilitation (CDCR), we developed and internally validated a 2-year mortality prediction model among incarcerated adults in the California prison system.

## METHODS

The study’s reporting was guided by the Transparent Reporting of a multivariable prediction model for Individual Prognosis Or Diagnosis + Artificial Intelligence (TRIPOD + AI) statement (eTable 1).^16^ Given their specialized knowledge of the prison healthcare system, we collaborated with CDCR physicians during weekly virtual meetings throughout all phases of the project, including cohort construction, predictor selection, model development, and model evaluation, aiming to develop a model with direct clinical utility.

### Study Design and Population

We used data from the CDCR, which manages the state of California’s prison system. We constructed a cohort of individuals aged 18 years and older who were in a CDCR prison for at least 1 year on 2/1/2018. We excluded individuals in prison for ≤ 1 year, as our primary goal was to identify longer-term incarcerated individuals with more detailed information on health conditions for accurate prognostication. We also excluded individuals in a hospice unit on February 1, 2018 (*N* = 10) as these individuals had already been identified as having limited life expectancy. We used a 1-year look-back period to obtain information on comorbidities and healthcare utilization.

### Outcomes

The primary outcome was 2-year mortality due to natural causes. Information on mortality was collected from 2/1/2018 (cohort start date) until 2/1/2020. We ended follow-up in February 2020 to avoid the early part of the COVID-19 pandemic, which contributed to many deaths in correctional settings.^17^ The CDCR performs mortality reviews for every death that occurs within the prison system, assigning each death to specific categories: accidental injury by others or to self, drug overdose, homicide by inmates/others, natural-expected, natural-unexpected, and suicide. We only included deaths due to natural causes (expected or unexpected), as our primary goal was to flag individuals for end-of-life services. There were 13 individuals in our cohort discharged through compassionate release during the study period. As CDCR does not collect death dates once discharged, we imputed a date of death 6 months after their discharge date based on discussions with CDCR physicians.

### Model Predictors

Candidate predictor variables were identified through systematic reviews and discussions with CDCR physicians.^15,18^ Detailed definitions are provided in eTable 2. Briefly, this included age (categorized into groups of < 40, 40–49, 50–59, 60–69, 70–79, and 80 +), sex (male vs. female), functional status (severe mobility restrictions with full-time wheelchair accommodation, severe mobility restrictions with intermittent wheelchair use, and other mobility restrictions (e.g., use of a walker)) and housing level on 2/1/2018 (general population housing, lower acuity infirmary beds (e.g., needing some assistance with activities of daily living), and higher acuity infirmary beds (e.g., needing more supervised health care provided by nursing staff)). Healthcare utilization included hospitalizations (0, 1, 2 +), intensive care unit (ICU) admissions, and dialysis use in the past year. Finally, we identified 78 comorbidities according to CDCR definitions, including diabetes, cancer, liver disease, and pulmonary fibrosis.

### Model Development

We used Cox proportional hazards regression to develop the model with a focus on predicting natural death at 2 years. Individuals were censored either on the date that they were released from a CDCR prison if their release occurred before 2 years, at 2 years if they stayed in a prison for the entire study period, or on the date that they experienced a non-natural death (e.g., homicide or suicide; *N* = 204). Based on sample size calculations (eMethods) and due to the large number of CDCR chronic conditions, we first removed comorbidities that had very low prevalence (< 1%) and contributed to ≤ 10 deaths. This resulted in 31 comorbidities in the pre-specified model. To reduce the number of comorbidities, we performed variable selection using Least Absolute Shrinkage and Selection Operator (LASSO). To increase the face validity of the model after discussions with CDCR physicians, we used a constrained version of LASSO to only include comorbidities with a positive coefficient (e.g., associated with increased hazard of mortality). We forced the other pre-specified variables into the model (age, sex, housing status, functional status, hospitalizations in the past year, ICU use, dialysis).

### Model Evaluation

We assessed model performance through discrimination and calibration.^19-21^ Discrimination was assessed with the concordance statistic (c-statistic), the time-specific area under the receiver operating characteristic curve (AUC) at 2 years, and the area under the precision-recall curve (AUPRC) at 2 years.^20^ Calibration was assessed at the 2-year timepoint by the calibration intercept (ideal value of 0), calibration slope (ideal value of 1), integrated calibration index (Eavg, average absolute difference between the loess predicted risk line and the 45 degree line; ideal value of 0), and visually with calibration plots.^22^ We calculated sensitivity, specificity, positive predictive value, and negative predictive value at four mortality risk thresholds chosen based on clinical judgment (5%, 10%, 20%, 60%).

We conducted two secondary analyses. First, we compared the performance of the Cox model to 3 variations of logistic regression. Second, we assessed model discrimination, calibration, and fairness metrics (equal opportunity, predictive parity, and disparate impact) across subgroups by race, ethnicity, and sex and in models with and without race and ethnicity as a predictor (eMethods). In general, fairness ranges for equal opportunity difference (whether the true positive rate is the same across subgroups), predictive parity (whether the positive predictive value is the same across subgroups), and disparate impact (ratio of the proportions of positive predictions between groups) are (−0.1, 0.1), (−0.1, 0.1), and (0.8, 1.25), respectively.^23-25^

We assessed internal validity via bootstrapping with 250 samples to quantify optimism in model performance.^26^ To present the final model, we provide the coefficients and baseline survival at 2 years. Finally, as a preliminary exploration of how the model may be used, the model formula was applied to the CDCR reporting data warehouse to identify currently incarcerated individuals at 20% and > 60% 2-year mortality risk. Two CDCR physicians reviewed the charts of 50 randomly selected individuals with 2-year mortality risk of 20% (*N* = 25) and > 60% (*N* = 25) to determine hospice and compassionate release eligibility. The study was reviewed and approved by the University of California, San Francisco Committee on Human Research and CDCR Research Oversight Committee. Statistical analyses were performed using R version 4.4.2 (R Project for Statistical Computing).

## RESULTS

### Cohort Characteristics

The initial cohort included 121,208 individuals ≥ 18 years in a CDCR prison on 2/1/2018 (eFigure 1). After excluding individuals incarcerated for ≤ 1 year on 2/1/2018 (*N* = 31,768) and those residing in a hospice bed on 2/1/2018 (*N* = 10), the final cohort consisted of 89,430 individuals. Overall, 9,153 (10.2%) were aged ≥ 60, 84,999 (95.0%) were male, 27,383 (30.6%) identified as Black, 37,047 (41.4%) identified as Hispanic, and 18,497 (20.7%) identified as White (Table 1). In comparison to California, the national prison population is similarly male predominant with a higher proportion of White adults (~ 31%) and lower proportion of Hispanic adults (~ 23%).^27,28^ There were 9,603 (10.7%) hospitalized at least once in the previous year, 7,520 (8.4%) with diabetes, and 2,609 (2.9%) with cancer.

After excluding deaths due to non-natural causes (e.g., homicide), we included 493 natural deaths (expected or unexpected) and 13 presumed deaths following compassionate release for a total of 506 deaths at 2 years (0.57%) (eTable 3). During the 2-year study period, 24,956 individuals were either released from a CDCR prison before 2/1/2020 or experienced a non-natural death (*N* = 204). Individuals with a natural death at 2-years tended to be older, have greater functional impairment, reside in a medical infirmary unit, have more hospitalizations, and have more chronic conditions. Median follow-up time was 2 years (interquartile range 1.9–2.0), and mean follow-up time was 1.7 years.

### Model Development

eTable 4 shows the unadjusted hazard ratios for predictors in the fully pre-specified model. Table 2 displays the multivariable-adjusted hazard ratios for variables in the constrained LASSO-Cox model. As expected, increased age, severe mobility restrictions, housing in medical infirmary units, higher number of hospitalizations, and chronic conditions were associated with increased hazard of mortality.

### Model Evaluation

**Figure 1:**
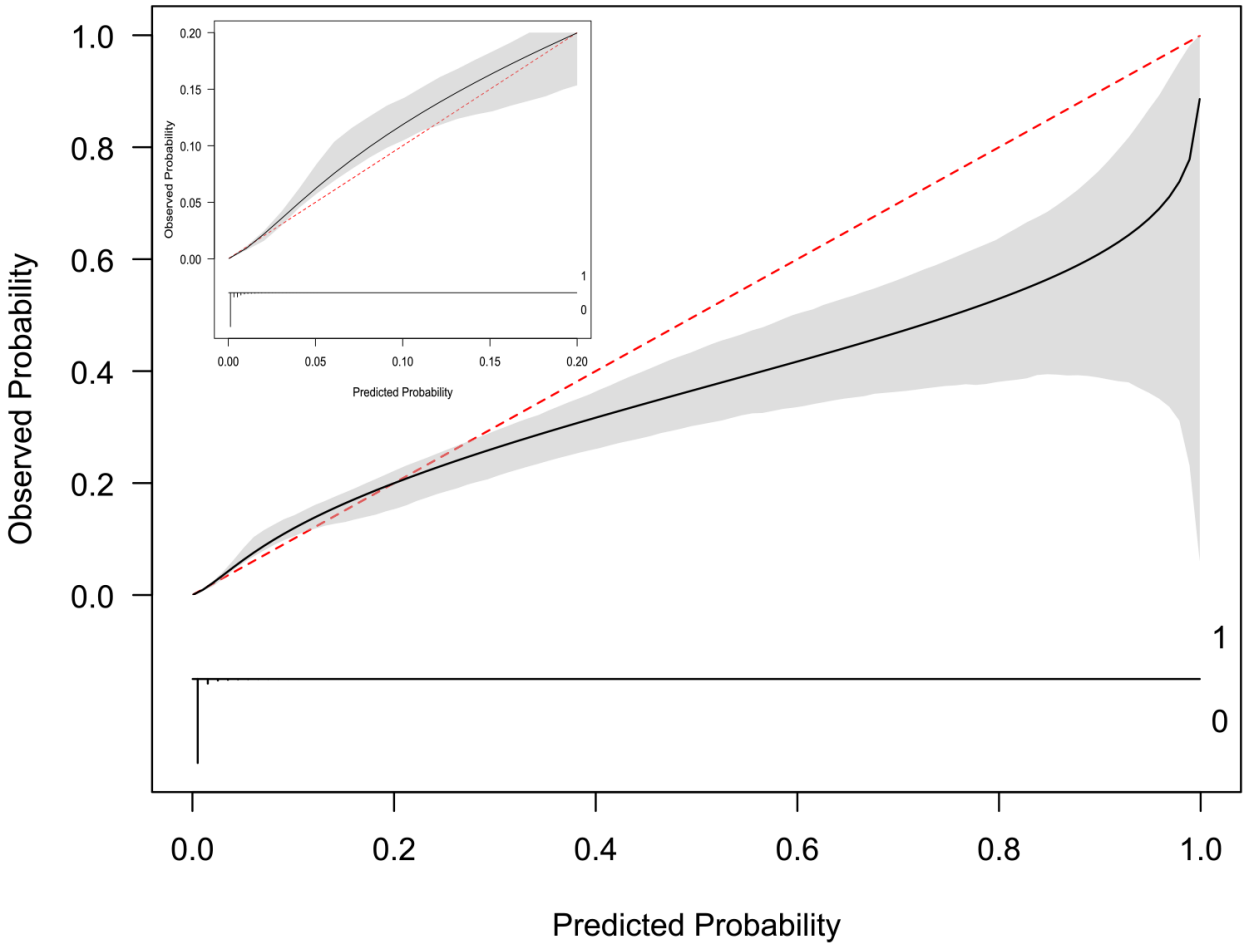
Calibration plot for the mortality prediction model at the 2-year timepoint. Note: The calibration plot indicates the agreement between the predicted mortality risk using the prediction model and observed probability. Perfect predictions would be at the 45-degree red dotted line. The grey bands indicate the 95% confidence limits of the calibration line. The x-axis of the larger plot spans from 0 to 1, and the x-axis of the smaller plot in the top left spans from 0 to 0.20 as the majority of individuals were at very low risk of 2-year mortality (< 2%).

The c-statistic and 2-year AUC prior to internal validation were 0.929 (95% CI 0.918–0.940) and 0.928 (95% CI 0.917–0.939), respectively (Table 3). After bootstrap internal validation, the optimism-corrected c-statistic and 2-year AUC were 0.926 (95% CI 0.915–0.938) and 0.926 (95% CI 0.915–0.938), respectively. At the 2-year timepoint, the model had good calibration at risk levels between 0–40% and modest over-estimation of risk at risk levels > 40% where the sample size of individuals was very low (Fig. 1). The optimism-corrected calibration slope was 0.9676, intercept was 0.0690, and integrated calibration index was 0.0014. The AUPRC was 0.188 (eFigure 2).

Table 4 and eFigure 3 show the model’s sensitivity, specificity, positive predictive value, and negative predictive at different risk thresholds. At a > 5% 2-year mortality risk threshold, the sensitivity was 47.6% (95% CI = 42.3–51.6%) and positive predictive value was 16.7% (95% CI = 14.3–18.5%).

### Secondary Analyses

Model performance for the LASSO-Cox model was similar to the full Cox model and alternative modeling approaches using logistic regression (eTables 5-6). Model performance was similar in models with and without race and ethnicity and within subgroups by race and ethnicity (eTable 7). Performance on fairness metrics, including equal opportunity difference, disparate impact, and predictive parity, were generally within commonly accepted fairness ranges (eTable 8). Model sensitivity at a 5% 2-year mortality threshold was lower among Hispanic individuals (40.1%, 95% CI = 31.6–48.7%) compared to non-Hispanic White individuals (51.1%, 95% CI = 43.4–58.7%) and Black individuals (49.0%, 95% CI = 38.4–59.6%).

### Presentation of the Model

The formula for the LASSO-Cox model and an example calculation are provided in eMethods. eTable 9 shows baseline characteristics for 10 individuals by predicted 2-year mortality risk.

### Exploratory Implementation

The model was applied to the CDCR reporting data warehouse for pilot implementation in March 2025, and chart reviews were conducted on 25 individuals with 20% 2-year mortality risk and 25 individuals with > 60% 2-year mortality risk. Among the 25 individuals with > 60% 2-year mortality risk, CDCR physicians identified 4 individuals who they felt were prognostically eligible for hospice (i.e., life-limiting illness with a prognosis of ≤ 6 months based on chart-review assessment of clinical factors) and had not previously engaged in end-of-life care discussions. Additionally, they identified 14 individuals who may be eligible for compassionate release (with only 2 having previously been referred). Among the 25 individuals with 20% 2-year mortality risk, they identified 4 individuals who they felt would be hospice eligible and 7 individuals who may be eligible for compassionate release (with only 1 having previously been referred).

## DISCUSSION

We developed and internally validated a 2-year mortality risk prediction model among incarcerated adults in the California prison system using predictors such as demographics, housing type, functional status, healthcare utilization, and comorbidities. The model can be used within the CDCR to generate a list of individuals at high mortality risk for advance care planning discussions, palliative care and hospice referrals, and consideration of compassionate release.

Several mortality prediction models have been developed across a variety of contexts and are available on websites such as ePrognosis.^15,18,29^ For example, the Lee index predicts 4- and 10-year mortality for community-dwelling adults aged 50 years and older.^30^ Incarcerated individuals develop chronic health conditions and experience physical and cognitive impairments at earlier ages compared to community-dwelling adults.^3,4^ To our knowledge, no prognostic models have been published for incarcerated adults who represent a particularly vulnerable population with unique environmental circumstances and increasingly complex healthcare needs. Our model included many factors associated with increased mortality in prior studies, including increased age, chronic conditions, and hospitalizations.^15^ We additionally relied on variables unique to the prison system, including housing in infirmary beds (i.e., due to functional impairments requiring nursing support) and the Disability Placement Program as a proxy of functional status.

Our model performed better than many mortality risk prediction models in terms of its discrimination (2-year AUC of 0.926). However, the inclusion of a large number of individuals at very low risk naturally led to very high discrimination, as it was easier to discriminate between individuals who did and did not die at follow-up.^31^ Reassuringly, our model showed acceptable performance in terms of its AUPRC, sensitivity, and positive predictive value at relevant thresholds.

We collaborated with CDCR physicians to develop this model, with the goal that physician leaders at CDCR would use the model at different thresholds of predicted mortality risk (e.g., 5%, 20%, or 60%) to generate lists of individuals to review for consideration of advance care planning, palliative care referrals, and compassionate release programs. While palliative care is significantly underutilized in large part due to lack of resources, other factors such as low staff retention and lack of knowledge around end-of-life care also likely play a significant role.^32,33^ A list of individuals at high mortality risk provided to healthcare teams would better allow staff to both identify and prioritize individuals likely to benefit most from these interventions.

Our model can also be used to identify individuals who may be eligible for compassionate release. These programs have historically been underutilized. In the state of California, between January 2015 and April 2021, only 53 individuals were released through the compassionate release program.^34^ Assembly Bill 960, which passed in September 2022, changed the program eligibility requirements in California to allow for individuals with a serious and advanced illness with an end-of-life trajectory or significant and permanent functional impairments to be considered for compassionate release.^35^ While this has led to a marked increase in the number of individuals discharged through compassionate release, some eligible individuals may not have been identified.^36^ To address this, CDCR has begun to systematically screen older adults and those in higher acuity infirmary beds. Based on our exploratory implementation and chart reviews, use of this prognostic model may identify additional individuals for review.

Most incarcerated individuals are from racial and ethnic minority groups and have faced accelerated aging due in part to the effects of systemic racism throughout the life course.^37,38^ We conducted assessments to evaluate differences in predictive performance among various subgroups and considered whether to include race and ethnicity as predictors.^39-42^ We ultimately chose not to include race and ethnicity as a predictor given similar model performance regardless of its inclusion and as it may reinforce harmful stereotypes and risks misinterpretation.^43^ Additionally, our model without race and ethnicity showed acceptable performance on fairness metrics based on commonly accepted ranges.^23,24,44^

### Strengths and Limitations

This study has several strengths. We included all individuals within the California prison system to develop a mortality model, which showed good discrimination and calibration. We collaborated with CDCR physicians to ensure the model was clinically relevant and could be readily integrated within the CDCR data warehouse to obtain prognostic estimates.

Our study has a few limitations. First, we specifically used a cohort of individuals from 2/1/2018 to 2/1/2020, as the early COVID-19 pandemic dramatically increased mortality within the California prison system. In more recent years, COVID-19 has had a smaller impact on mortality. We plan to track the model’s performance over time and, if necessary, update it based on more recent trends in mortality.^45^ Second, our model was specifically developed among individuals in the California prison system and may not apply to incarcerated individuals in other states. Future studies evaluating its performance in other geographic settings are needed.

## CONCLUSION

Our 2-year mortality prediction model for adults within the California prison system performed well on measures of discrimination, calibration, and classification. The model can be used to flag individuals at higher risk for mortality for consideration of advance care planning interventions, palliative care and hospice referrals, and compassionate release.

## Supplementary Material

11606_2025_10103_MOESM1_ESM

**Supplementary Information** The online version contains supplementary material available at https://doi.org/10.1007/s11606-025-10103-w.

## Figures and Tables

**Table 1:** Baseline Characteristics of Incarcerated Individuals who were in a California Prison for at Least 1 Year on February 1, 2018

Characteristic	Overall cohort (*N* = 89,430)	Natural death at 2 years (*N* = 506, 0.57%)*
Age in years, median (IQR)	40 (20)	64 (14)
Age group		
< 40	43050 (48.1%)	13 (2.6%)
40–49	20753 (23.2%)	42 (8.3%)
50–59	16474 (18.4%)	136 (26.9%)
60–69	7163 (8.0%)	179 (35.4%)
70–79	1734 (1.9%)	97 (19.2%)
80 +	256 (0.3%)	39 (7.7%)
Sex^†^		
Male	84999 (95.0%)	489 (96.6%)
Female	4431 (5.0%)	17 (3.4%)
Race and ethnicity^‡^		
American Indian, Alaskan Native, Asian, or Pacific Islander	2087 (2.3%)	14 (2.8%)
Non-Hispanic Black	27383 (30.6%)	127 (25.1%)
Hispanic	37047 (41.4%)	131 (25.9%)
Non-Hispanic White	18497 (20.7%)	203 (40.1%)
Other/Unknown	4416 (4.9%)	31 (6.1%)
Disability Placement Program (DPP) category^§^		
Severe mobility restrictions with full-time wheelchair use	647 (0.7%)	55 (10.9%)
Severe mobility restrictions with intermittent wheelchair use	1069 (1.2%)	85 (16.8%)
Other mobility restriction (e.g., assistive device other than wheelchair)	5615 (6.3%)	138 (27.3%)
Housing level		
General housing	83510 (93.4%)	327 (64.6%)
Other housing^∥^	4767 (5.3%)	11 (2.2%)
Lower acuity infirmary bed^¶^	616 (0.7%)	55 (10.9%)
Higher acuity infirmary bed^¶^	537 (0.6%)	113 (22.3%)
Hospitalizations in the previous year		
0	79827 (89.3%)	259 (51.2%)
1	6943 (7.8%)	106 (20.9%)
2 +	2660 (3.0%)	141 (27.9%)
Any ICU admission in the previous year	154 (0.2%)	13 (2.6%)
Dialysis use	97 (0.1%)	< 10 (< 1%)
Chronic conditions		
Aplastic anemia	62 (0.1%)	11 (2.2%)
Cancer	2609 (2.9%)	173 (34.2%)
Chronic pain	13609 (15.2%)	217 (42.9%)
Chronic kidney disease	16340 (18.3%)	304 (60.1%)
Chronic obstructive pulmonary disease	3417 (3.8%)	148 (29.2%)
Arrhythmia	2153 (2.4%)	105 (20.8%)
Congestive heart failure	824 (0.9%)	67 (13.2%)
Peripheral vascular disease	1056 (1.2%)	63 (12.5%)
Thromboembolic disease	703 (0.8%)	41 (8.1%)
Diabetes	7520 (8.4%)	169 (33.4%)
End stage liver disease	1661 (1.9%)	112 (22.1%)
Hepatitis C	17825 (19.9%)	182 (36.0%)
Immunosuppressed	1157 (1.3%)	49 (9.7%)
Ostomy	183 (0.2%)	13 (2.6%)
Pulmonary fibrosis	84 (0.1%)	10 (2.0%)
Unspecified hepatitis	1087 (1.2%)	20 (4.0%)

*Abbreviations*: ICU, intensive care unit

* The number of natural deaths (*N* = 506) represents 493 natural deaths during the 2-year study period in addition to 13 presumed deaths from individuals in our cohort who were released from the prison system through compassionate release during the 2-year study period

† This variable included the categories of male, female, and non-binary. Female (*N* = 3,945) and non-binary (*N* = 486) categories were combined into a single category

‡ Information on race and ethnicity was obtained from the California Department of Corrections and Rehabilitation race category variable which is mostly based on self-reported data. The Hispanic category combines the categories of Mexican, Cuban, and Hispanic within the CDCR data

§ The Disability Placement Program classifies individuals based on mobility restrictions. See Supplement Table 2 in the Supplement for additional details

∥ This category includes individuals in restricted housing units and mental health housing levels

¶ In the California Department of Corrections and Rehabilitation, higher acuity infirmary beds are termed correctional treatment centers (CTC), providing care to individuals who need professionally supervised health care. Lower acuity infirmary beds are termed outpatient housing units (OHU), providing care to individuals who may need some assistance, which places them at personal or security risk in the general population

**Table 2:** Multivariable-adjusted Hazard Ratios for Variables Included in the LASSO-Cox Mortality Prediction Model Among Incarcerated Individuals*

Characteristic	Adjusted hazard ratio (95% CI)	Beta-coefficient (95% CI)	*P*-value
Age group			
< 40	0.05 (0.03, 0.08)	−3.07 (−3.66, −2.47)	< 0.001
40–49	0.23 (0.16, 0.33)	−1.46 (−1.82, −1.10)	< 0.001
50–59	0.63 (0.50, 0.81)	−0.46 (−0.70, −0.22)	< 0.001
60–69	Ref	Ref	
70–79	1.32 (1.01, 1.72)	0.28 (0.01, 0.54)	0.039
80 +	2.50 (1.70, 3.68)	0.92 (0.53, 1.30)	< 0.001
Sex			
Male	Ref	Ref	
Female	0.83 (0.51, 1.35)	−0.19 (−0.68, 0.30)	0.45
Disability Placement Program (DPP) category^†^			
Severe mobility restrictions with full-time wheelchair use	1.42 (0.97, 2.09)	0.35 (−0.03, 0.73)	0.073
Severe mobility restrictions with intermittent wheelchair use	2.01 (1.46, 2.76)	0.70 (0.38, 1.02)	< 0.001
Other mobility restriction (e.g., assistive device other than wheelchair)	1.51 (1.19, 1.93)	0.42 (0.17, 0.66)	0.001
None	Ref	Ref	
Housing level			
General and other housing^‡^	Ref	Ref	
Lower acuity infirmary bed^§^	2.33 (1.68, 3.22)	0.85 (0.52, 1.17)	< 0.001
Higher acuity infirmary bed^§^	4.51 (3.33, 6.10)	1.51 (1.20, 1.81)	< 0.001
Hospitalizations in the previous year			
0	Ref	Ref	
1	1.95 (1.53, 2.47)	0.67 (0.43, 0.90)	< 0.001
2 +	2.52 (1.95, 3.25)	0.92 (0.67, 1.18)	< 0.001
Any intensive care unit admission in the previous year			
Yes	1.13 (0.63, 2.02)	0.12 (−0.47, 0.70)	0.69
No	Ref	Ref	
Dialysis			
Yes	1.18 (0.53, 2.64)	0.17 (−0.63, 0.97)	0.68
No	Ref	Ref	
Chronic conditions			
Aplastic anemia	2.34 (1.25, 4.41)	0.85 (0.22, 1.48)	0.008
Cancer	3.17 (2.58, 3.89)	1.15 (0.95, 1.36)	< 0.001
Chronic pain	1.14 (0.94, 1.39)	0.13 (−0.06, 0.33)	0.18
Chronic kidney disease	1.27 (1.03, 1.55)	0.24 (0.03, 0.44)	0.024
Chronic obstructive pulmonary disease	1.16 (0.93, 1.45)	0.15 (−0.07, 0.37)	0.19
Arrhythmia	1.28 (0.98, 1.66)	0.24 (−0.02, 0.51)	0.068
Congestive heart failure	1.08 (0.79, 1.49)	0.08 (−0.24, 0.40)	0.63
Peripheral vascular disease	1.06 (0.79, 1.41)	0.05 (−0.24, 0.35)	0.71
Thromboembolic disease	1.16 (0.82, 1.65)	0.15 (−0.20, 0.50)	0.40
Diabetes	1.19 (0.98, 1.45)	0.18 (−0.02, 0.37)	0.083
End stage liver disease	2.65 (2.05, 3.43)	0.98 (0.72, 1.23)	< 0.001
Hepatitis C	1.12 (0.91, 1.39)	0.12 (−0.10, 0.33)	0.28
Immunosuppressed	1.30 (0.94, 1.79)	0.26 (−0.06, 0.58)	0.11
Ostomy	1.73 (0.97, 3.07)	0.55 (−0.03, 1.12)	0.061
Pulmonary fibrosis	2.09 (1.08, 4.03)	0.74 (0.08, 1.39)	0.028
Unspecified hepatitis	1.37 (0.86, 2.18)	0.32 (−0.15, 0.78)	0.18

*Abbreviations*: *CI*, confidence interval; Ref, reference group

* The baseline survival at 2-years was 0.9936. For reference groups, the hazard ratio is 1 and beta-coefficient is 0

† The Disability Placement Program classifies individuals based on mobility restrictions. See Supplement Table 2 in the Supplement for additional details

‡ This category includes individuals in general housing and other housing units (e.g., restricted housing units and mental health housing levels)

§ In the California Department of Corrections and Rehabilitation, higher acuity infirmary beds are termed correctional treatment centers (CTC), providing care to individuals who need professionally supervised health care. Lower acuity infirmary beds are termed outpatient housing units (OHU), providing care to individuals who may need some assistance which places them at personal or security risk in the general population

**Table 3:** Performance of the LASSO-Cox Mortality Prediction Model Among Incarcerated Individuals

Performance metric	Apparent performance (95% CI)	Optimism corrected performance (95% CI)
c-statistic	0.929 (0.918, 0.940)	0.926 (0.915, 0.938)
Time-specific AUC at 2 years	0.928 (0.917, 0.939)	0.926 (0.915, 0.938)
Calibration intercept	0.0 (0.0, 0.0)	0.0690 (0.0689, 0.0692)
Calibration slope	1.0 (1.0, 1.0)	0.9676 (0.9674, 0.9677)
Integrated calibration index (Eavg)	0.0013 (0.0009, 0.0018)	0.0014 (0.0010, 0.0018)

*Abbreviations*: *AUC*, area under the receiver operating characteristic curve; c-statistic, concordance statistic

The c-statistic quantifies the ability of the model to rank individuals based on the predicted risk of experiencing the outcome. The time-specific AUC at 2 years quantifies the ability of the model to rank individuals at the 2-year time point. Values for both range from 0.5 to 1.0 (perfect discrimination). The calibration intercept quantifies whether predicted probabilities are, on average, too high or too low. The ideal value for the intercept is 0, and for the model’s apparent performance is 0. The calibration slope quantifies whether predicted probabilities are too extreme or modest. The ideal value is 1, and the model’s apparent performance is 1. The integrated calibration index is a weighted average of the absolute difference between predicted probabilities and probabilities derived from a smooth calibration curve. The ideal value is 0

**Table 4:** Performance Metrics at Different 2-Year Mortality Risk Thresholds Among 89,430 Individuals in the California Prison System

2-year mortality risk threshold	Number of individuals above threshold	Deaths identified (out of 506 deaths)	Sensitivity (95% CI)	Specificity (95% CI)	Positive predictive value (95% CI)	Negative predictive value (95% CI)
> 5%	1558	244	47.6% (42.3%, 51.6%)	98.4% (98.2%, 98.4%)	16.7% (14.3%, 18.5%)	99.7% (99.6%, 99.7%)
> 10%	773	168	32.5% (28.1%, 36.7%)	99.3% (99.1%, 99.4%)	23.4% (20.4%, 26.8%)	99.6% (99.5%, 99.6%)
> 20%	355	103	19.9% (16.6%, 23.4%)	99.7% (99.7%, 99.7%)	30.2% (25.4%, 35.4%)	99.5% (99.4%, 99.5%)
> 60%	62	30	5.6% (3.8%, 7.4%)	99.9% (99.9%, 99.9%)	50.6% (37.2%, 63.9%)	99.4% (99.3%, 99.4%)

*Abbreviations*: *CI*, confidence interval

In this table, the number of individuals above the specific thresholds and deaths identified at specific thresholds were obtained from a single sample. The values for sensitivity, specificity, positive predictive value, and negative predictive value were obtained through bootstrapped samples and do not precisely correspond to calculations based on a single sample

## Data Availability

The data supporting the findings of this study were obtained from the California Department of Corrections and Rehabilitation reporting data warehouse. Use of this data requires approval from the Research Oversight Committee and receipt of a data use agreement.
