# Supplementary material for: Development of a Mortality Prediction Model for Incarcerated Adults to Identify Palliative Care Needs
Source: J Gen Intern Med. Author manuscript; Available in PMC 2026 Apr 13. (PMC13075380; doi:10.1007/s11606-025-10103-w)
Supplement: 11606_2025_10103_MOESM1_ESM [file NIHMS2158757-supplement-11606_2025_10103_MOESM1_ESM.docx]

**Supplementary Materials**

**Supplementary Methods**

**eFigure 1**: Flow diagram for cohort construction and assessment of natural deaths

**eFigure 2:** Area under the precision recall curve for the LASSO Cox regression model for predicting 2-year mortality

**eFigure 3:** Comparison of sensitivity, specificity, positive predictive value, and negative predictive value at 2-year mortality risk thresholds from 0 to 60% for the LASSO Cox model

**eTable 1:** TRIPOD+AI Checklist

**eTable 2:** Full list of predictors included in the prognostic model with detailed description and specification

**eTable 3:** Classification of deaths during the 2-year study period from February 1, 2018 to February 1, 2020

**eTable 4:** Univariable association between predictors and 2-year mortality

**eTable 5:** Comparison in model performance among the modeling approaches in predicting 2-year mortality for incarcerated individuals

**eTable 6:** Performance metrics at different 2-year mortality risk thresholds for the modeling approaches

**eTable 7**: Model performance and fairness evaluation for a LASSO Cox model with and without race and ethnicity at a threshold of 5% 2-year mortality risk

**eTable 8:** Model performance and fairness evaluation for a LASSO Cox model within subgroups by race and ethnicity and sex at threshold of 5% 2-year mortality risk

**eTable 9:** Baseline characteristics for 10 randomly selected individuals based on predicted 2-year mortality risk

**Supplementary Methods**

**Additional information on candidate predictors**

Candidate predictors included demographic factors such as age (categorized into groups of <40, 40-49, 50-59, 60-69, 70-79, and 80+) and sex (male vs. female). The California Department of Corrections and Rehabilitation (CDCR) has a Disability Placement Program (DPP), which helps place inmates with disabilities in appropriate housing and programs. As a measure of functional status, we categorized individuals based on DPP codes. This included: severe mobility restrictions that require full-time wheelchair accommodation, severe mobility restrictions but only using a wheelchair intermittently, and other mobility restrictions (e.g., use of an assistive device other than a wheelchair or requiring a relatively level terrain to ambulate). A final reference group was created for individuals not assigned a DPP code.

We categorized individuals based on the level of housing they were in on February 1, 2018. Categories included general population housing, lower acuity infirmary beds (CDCR’s outpatient housing units), and higher acuity infirmary beds (CDCR’s correctional treatment centers). Individuals in lower acuity infirmary beds may receive outpatient health services and assistance with activities of daily living, and individuals in higher acuity infirmary beds are typically in need of supervised health care beyond that normally provided in the community on an outpatient basis. We grouped some other housing types (e.g., restricted housing units) under the general population housing because they were not correlated with health status.

Previous healthcare utilization included hospitalizations in the past year (categorized as 0, 1, 2+) and an intensive care unit admission in the previous year. We also identified individuals who were on dialysis in the past year.

Finally, we identified 78 comorbidities according to CDCR definitions over the 1-year look-back period, including diabetes, cancer, liver disease, and pulmonary fibrosis. CDCR has specific definitions to identify individuals with chronic conditions. For example, individuals with cancer are identified based on an active electronic health record diagnosis related to cancer (based on International Classification of Diseases (ICD) or Systematized Nomenclature of Medicine (SNOMED) codes), a hospitalization or specialty visit claim with a primary diagnosis code related to cancer, or an active prescription for cancer medication.

**Sample size calculation**

We used the pmsampsize package in Stata to calculate the minimum sample size to ensure key parameters were estimated precisely and to minimize overfitting. Since no previously published models in this population were identified, we used a c-statistic of 0.75 which is typical of most mortality prediction models among community-dwelling adults. The pmsampsize package requires a Cox-Snell R squared value which can be estimated from the c-statistic. Using simulation, we estimated the Cox-Snell R squared value to be 0.005. With 0.9 level of shrinkage, 0.005 as the Cox-Snell R squared value, 2-year follow-up time, 1.7 mean follow-up time in our cohort, and prevalence of 2-year natural death of 0.0057, the minimum sample size would be 82,000 individuals. Therefore, our cohort size of 89,430 was felt to be sufficient.

**Modeling approaches**

Given that we did not have information on mortality if an individual was released from a CDCR prison (e.g., either on parole or following completion of their sentence), we chose Cox proportional hazards regression as a primary analysis. This approach accounts for loss to follow-up during the 2-year study period. In the primary Cox regression model, we considered individuals to have the event is they experienced a natural death during the 2-year study period. Individuals were censored either on the date that they were released from a CDCR prison if their release occurred before 2 years, at 2 years if they stayed in a prison for the entire study period, or on the date that they experienced a non-natural death (e.g., homicide or suicide; N=204).

In our cohort of 89,430 individuals who were incarcerated for at least 1 year in a CDCR prison on February 1, 2018, 88,924 individuals did not experience a natural death during the 2-year study period. Of these 88,924 individuals, 24,956 were either released from a CDCR prison before February 1, 2020 (end date of the 2-year study period) or experienced a non-natural death (e.g., homicide; N=204). Of the 24,956 people, 2,100 returned to a CDCR prison and were still alive on February 1, 2020.

As secondary analyses, we decided to look at model performance using logistic regression with a primary outcome of 2-year mortality. In the first approach, we performed logistic regression whereby we considered individuals to not have the event (natural death at 2-years) if they were either alive in a prison on February 1, 2020, were released before February 1, 2020, or experienced a non-natural death (e.g., homicide). In the second approach, we performed logistic regression adding a variable indicating the amount of exposure time during the study period. Individuals who were alive in a prison on February 1, 2020 were assigned an exposure time of 2 years. Individuals who were released prior to February 1, 2020 were assigned an exposure time equal to the date of their release minus the start date of the study (February 1, 2018). Individuals who experienced a non-natural death were assigned an exposure time equal to the date of their non-natural death minus the start date of the study. In the third approach, we performed logistic regression adding a variable indicating the amount of exposure time during the study period and allowed for additional time to accrue if individuals returned to a prison after being released. For example, if an individual were released on parole on June 1, 2018, returned to a prison on January 1, 2020, and remained alive in a prison on February 1, 2020, we added up their total time in a prison (from February 1, 2018 to June 1, 2018 and from January 1, 2020 to February 1, 2020).

**Model fairness assessment**

Several studies have raised concerns that prognostic models may perpetuate healthcare disparities. This may occur if, for example, there are differences in model performance with respect to certain demographic features, such as race, ethnicity, age, and sex. Algorithmic fairness is focused on designing algorithms that minimize bias and ensure equitable treatment for all individuals and groups. To this end, we addressed algorithmic fairness in two primary ways: examining model performance and fairness metrics in (1) models with and without race/ethnicity as a predictor and (2) across subgroups by race/ethnicity and sex.

To examine model performance, we focused on discrimination (area under the receiver operating characteristic curve) and calibration (integrated calibration index). We additionally calculated several fairness metrics that have been proposed to evaluate model fairness. While there is no ”one size fits all” fairness metric and each provides a different perspective, we calculated 3 commonly used metrics: equal opportunity, disparate impact, and predictive parity. To calculate these metrics, we used a 2-year mortality risk threshold of >5% as this is one possible threshold that clinicians may use to identify individuals who may benefit from advance care planning.

The equal opportunity fairness metric evaluates whether the true positive rate (sensitivity) is the same across subgroups. Equal opportunity is defined as when, within each subgroup, there is an equal probability of an individual in a positive class (dies within 2 years) to have a positive prediction (e.g., 2-year mortality risk >5%). This prioritizes equal error rates across groups, and the target value for equal opportunity difference is 0. We prioritized this fairness metric given that our goal was to flag individuals for palliative care interventions that pose minimal harm. In other words, we prioritize the true positive rate (sensitivity) as the consequence of a false negative (missing a person at high mortality risk who could benefit from palliative care and advance care planning interventions) outweighs the consequences of false positives (flagging a patient as high risk when in actuality they were at low risk).

Disparate impact assesses the ratio of the proportions of positive predictions between the unprivileged and privileged groups. For example, based on disparate impact, the ratio of the proportions of individuals classified as high risk (2-year mortality >5%) in non-White and White groups would have a target value of 1.

Predictive parity assesses whether the precision rate (positive predictive value) is similar across groups. In the context of this study, precision refers to the proportion of individuals who were predicted as high risk (e.g., 2-year mortality >5%) who died at 2 years. Predictive parity difference for race/ethnicity in our study can be defined as the difference in positive predictive values between non-White groups (Black, Hispanic, Other) and White groups. The target value for predictive parity difference is 0.

Of note, many fairness criteria are mutually incompatible. Therefore, the choice of metric should be context-specific. As noted above, we chose to prioritize equal opportunity, which places a higher penalty on missing potential cases, given that the primary goal of our prognostic model is for screening.

**Model formula**

The formula for the final prediction model is as follows:

$$\frac{h_{i}\left( t \right)}{h_{0}\left( t \right)}=\exp\left( \beta_{1}Ageunder40+ \beta_{2}Age40to49+ \beta_{3}Age50to59+ \beta_{4}Age60to69+ \beta_{5}Age70to79+ \beta_{6}80andolder+ \beta_{7}Femalesex+ \beta_{8}Severemobilitywithwheelchair+ \beta_{9}Severemobilitywithintermittentwheelchair + \beta_{10}Othermobilityrestriction+ \beta_{11}Generalhouse + \beta_{12}Loweracuityinfirmary+ \beta_{13}Higheracuityinfirmary+ \beta_{14}nohospitalizations+ \beta_{15}1hospitalization+ \beta_{16}2ormorehospitalizations+ \beta_{17}ICUadmission+ \beta_{18}dialysis+ \beta_{19}aplasticanemia+ \beta_{20}cancer+ \beta_{21}chronicpain+ \beta_{22}chronickidneydisease+ \beta_{23}COPD+ \beta_{24}arrhythmia+ \beta_{25}heartfailure+ \beta_{26}peripheralvasculardisease+ \beta_{27}thromboembolicdisease+ \beta_{28}diabetes+ \beta_{29}endstageliverdisease+ \beta_{30}hepatitisC+ \beta_{31}immunosuppressed+ \beta_{32}ostomy+ \beta_{33}pulmonaryfibrosis+ \beta_{34}unspecifiedhepatitis \right)$$

where

$\frac{h_{i}\left( t \right)}{h_{0}\left( t \right)}$ : hazard ratio

$\beta_{1}, \beta_{2},\ldots,\beta_{19}$: regression coefficients

To obtain an individual’s mortality risk at a specific time t, use the formula:

$$1-{S_{o}(t)}^{exp(X_{i}\beta_{i})}$$

where $S_{o}(t)$ is the baseline survival probability at time t

and X_i_β_i_ = $\left( \beta_{1}Ageunder40+ \beta_{2}Age40to49+ \beta_{3}Age50to59+ \beta_{4}Age60to69+ \beta_{5}Age70to79+ \beta_{6}80andolder+ \beta_{7}Femalesex+ \beta_{8}Severemobilitywithwheelchair+ \beta_{9}Severemobilitywithintermittentwheelchair + \beta_{10}Othermobilityrestriction+ \beta_{11}Generalhouse + \beta_{12}Loweracuityinfirmary+ \beta_{13}Higheracuityinfirmary+ \beta_{14}nohospitalizations+ \beta_{15}1hospitalization+ \beta_{16}2ormorehospitalizations+ \beta_{17}ICUadmission+ \beta_{18}dialysis+ \beta_{19}aplasticanemia+ \beta_{20}cancer+ \beta_{21}chronicpain+ \beta_{22}chronickidneydisease+ \beta_{23}COPD+ \beta_{24}arrhythmia+ \beta_{25}heartfailure+ \beta_{26}peripheralvasculardisease+ \beta_{27}thromboembolicdisease+ \beta_{28}diabetes+ \beta_{29}endstageliverdisease+ \beta_{30}hepatitisC+ \beta_{31}immunosuppressed+ \beta_{32}ostomy+ \beta_{33}pulmonaryfibrosis+ \beta_{34}unspecifiedhepatitis \right)$

Beta-coefficients for all variables can be found in Table 2.

For estimating the 2-year mortality risk, the baseline survival probability at 2 years equals 0.9936.

As an example calculation, consider a 65-year-old man with severe mobility restrictions requiring full-time use of a wheelchair. He is currently residing in a higher-acuity infirmary bed and has had 3 hospitalizations in the past year. He has chronic pain, chronic kidney disease, chronic obstructive pulmonary disease, diabetes, and an ostomy. His predicted 2-year mortality is 30%.

**eFigure 1**: Flow diagram for cohort construction and assessment of natural deaths

Individuals 18 years and older in a CDCR prison on February 1, 2018

(121,208 individuals)

Exclude individuals not within a prison for at least 1-year prior to the index date (N=31,768)

Individuals 18 years and older in a CDCR prison on February 1, 2018 for at least 1 year

(89,440 individuals)

Exclude people who were in a hospice unit on February 1, 2018 (N=10)

Individuals 18 years and older in a CDCR prison on February 1, 2018 for at least 1 year not in a hospice bed (89,430 individuals)

Died at 2 years from any cause (N=697)

Discharged through compassionate release (N=13)

Exclude deaths not due to natural causes (N=204)

Died at 2 years from natural cause or presumed death due to compassionate release (N=506)

**eFigure 2:** Comparison of sensitivity, specificity, positive predictive value, and negative predictive value at 2-year mortality risk thresholds from 0 to 60% for the LASSO Cox model

**
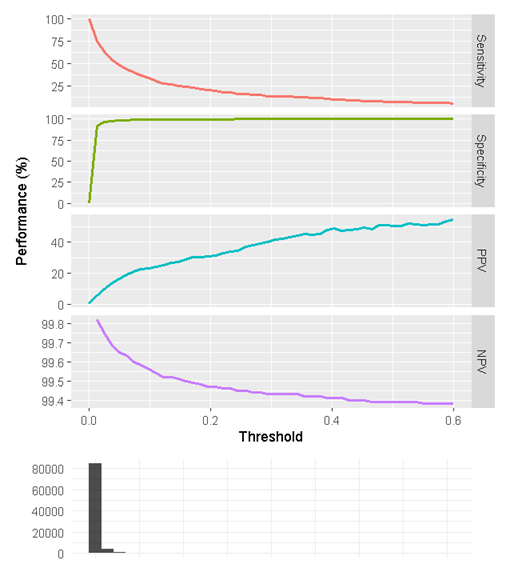
**

Abbreviations: NPV, negative predictive value; PPV, positive predictive value.

Note: The bottom panel shows a histogram of the predicted 2-year mortality risk for individuals in the cohort.

**eFigure 3:** Area under the precision recall curve for the LASSO Cox regression model for predicting 2-year mortality

**
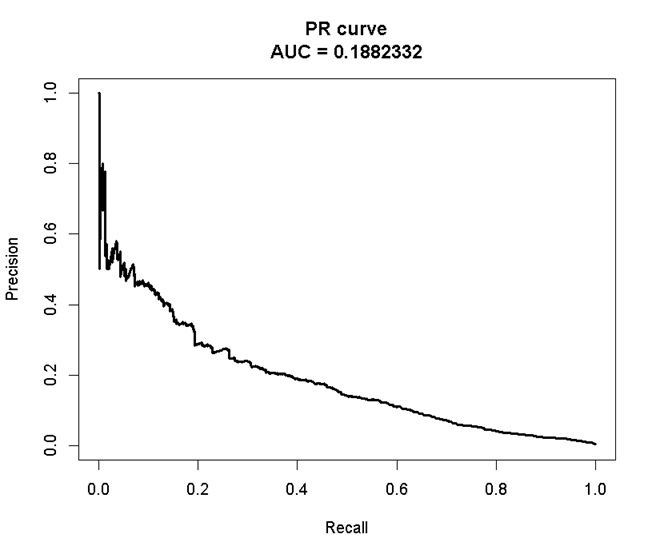
**

Note: A precision-recall curve shows the trade-off between precision (also known as positive predictive value) and recall (also known as sensitivity, or true positive rate) across different decision thresholds.

**eTable 1:** TRIPOD+AI Checklist

| **Section/Topic Item Development Checklist item**  **/ evaluation**^1^ | | | | **Reported on page** |
| --- | --- | --- | --- | --- |
| **TITLE** | | | |  |
| *Title* | 1 | D;E | Identify the study as developing or evaluating the performance of a multivariable prediction model, the target population, and the outcome to be predicted | 1 |
| **ABSTRACT** | | | | |
| *Abstract* | 2 | D;E | See TRIPOD+AI for Abstracts checklist | 3 |
| **INTRODUCTION** | | | | |
| *Background* | 3a | D;E | Explain the healthcare context (including whether diagnostic or prognostic) and rationale for developing or evaluating the prediction model, including references to existing models | 5 |
|  | 3b | D;E | Describe the target population and the intended purpose of the prediction model in the context of the care pathway, including its intended users (e.g., healthcare professionals, patients, public) | 6 |
|  | 3c | D;E | Describe any known health inequalities between sociodemographic groups | 5, 9 |
| *Objectives* | 4 | D;E | Specify the study objectives, including whether the study describes the development or validation of a prediction model (or both) | 6 |
| **METHODS** | | | | |
| *Data* | 5a | D;E | Describe the sources of data separately for the development and evaluation datasets (e.g., randomised trial, cohort, routine care or registry data), the rationale for using these data, and representativeness of the data | 6-7 |
|  | 5b | D;E | Specify the dates of the collected participant data, including start and end of participant accrual; and, if applicable, end of follow-up | 6-7 |
| *Participants* | 6a | D;E | Specify key elements of the study setting (e.g., primary care, secondary care, general population)  including the number and location of centres | 6-7 |
|  | 6b | D;E | Describe the eligibility criteria for study participants | 7 |
|  | 6c | D;E | Give details of any treatments received, and how they were handled during model development or evaluation, if relevant | N/A |
| *Data preparation* | 7 | D;E | Describe any data pre-processing and quality checking, including whether this was similar across  relevant sociodemographic groups | Supplementary Methods, eTable 2 |
| *Outcome* | 8a | D;E | Clearly define the outcome that is being predicted and the time horizon, including how and when assessed, the rationale for choosing this outcome, and whether the method of outcome assessment is  consistent across sociodemographic groups | 7 |
|  | 8b | D;E | If outcome assessment requires subjective interpretation, describe the qualifications and demographic characteristics of the outcome assessors | N/A |
|  | 8c | D;E | Report any actions to blind assessment of the outcome to be predicted | N/A |
| *Predictors* | 9a | D | Describe the choice of initial predictors (e.g., literature, previous models, all available predictors) and  any pre-selection of predictors before model building | 7-8 |
|  | 9b | D;E | Clearly define all predictors, including how and when they were measured (and any actions to blind assessment of predictors for the outcome and other predictors) | 7-8, eTable 2 |
|  | 9c | D;E | If predictor measurement requires subjective interpretation, describe the qualifications and demographic characteristics of the predictor assessors | eTable 2 |
| *Sample size* | 10 | D;E | Explain how the study size was arrived at (separately for development and evaluation), and justify that  the study size was sufficient to answer the research question. Include details of any sample size calculation | Supplementary Methods |
| *Missing data* | 11 | D;E | Describe how missing data were handled. Provide reasons for omitting any data | Supplementary Methods |
| *Analytical methods* | 12a | D | Describe how the data were used (e.g., for development and evaluation of model performance) in the analysis, including whether the data were partitioned, considering any sample size requirements | 8 |
|  | 12b | D | Depending on the type of model, describe how predictors were handled in the analyses (functional form,  rescaling, transformation, or any standardisation). | 8 |
|  | 12c | D | Specify the type of model, rationale^2^, all model-building steps, including any hyperparameter tuning,  and method for internal validation | 8 |
|  | 12d | D;E | Describe if and how any heterogeneity in estimates of model parameter values and model performance was handled and quantified across clusters (e.g., hospitals, countries). See TRIPOD-Cluster for  additional considerations^3^ | 9 |
|  | 12e | D;E | Specify all measures and plots used (and their rationale) to evaluate model performance (e.g., discrimination, calibration, clinical utility) and, if relevant, to compare multiple models | 9 |
|  | 12f | E | Describe any model updating (e.g., recalibration) arising from the model evaluation, either overall or for particular sociodemographic groups or settings | N/A |
|  | 12g | E | For model evaluation, describe how the model predictions were calculated (e.g., formula, code, object, application programming interface) | 9 |
| *Class imbalance* | 13 | D;E | If class imbalance methods were used, state why and how this was done, and any subsequent methods to  recalibrate the model or the model predictions | N/A |
| *Fairness* | 14 | D;E | Describe any approaches that were used to address model fairness and their rationale | 9, Supplementary Methods |
| *Model output* | 15 | D | Specify the output of the prediction model (e.g., probabilities, classification). Provide details and  rationale for any classification and how the thresholds were identified | 9-10 |

| *Training versus*  *evaluation* | 16 | D;E | Identify any differences between the development and evaluation data in healthcare setting, eligibility  criteria, outcome, and predictors | N/A |
| --- | --- | --- | --- | --- |
| *Ethical approval* | 17 | D;E | Name the institutional research board or ethics committee that approved the study and describe the participant-informed consent or the ethics committee waiver of informed consent | 10 |
| **OPEN SCIENCE** | | | | |
| *Funding* | 18a | D;E | Give the source of funding and the role of the funders for the present study | 16 |
| *Conflicts of interest* | 18b | D;E | Declare any conflicts of interest and financial disclosures for all authors | 16 |
| *Protocol* | 18c | D;E | Indicate where the study protocol can be accessed or state that a protocol was not prepared | N/A |
| *Registration* | 18d | D;E | Provide registration information for the study, including register name and registration number, or state  that the study was not registered | N/A |
| *Data sharing* | 18e | D;E | Provide details of the availability of the study data | Supplement 2 |
| *Code sharing* | 18f | D;E | Provide details of the availability of the analytical code^4^ | Supplement 2 |
| **PATIENT & PUBLIC INVOLVEMENT** | | | | |
| *Patient & Public Involvement* | 19 | D;E | Provide details of any patient and public involvement during the design, conduct, reporting, interpretation, or dissemination of the study or state no involvement. | N/A (collaborated with CDCR physicians) |
| **RESULTS** | | | | |
| *Participants* | 20a | D;E | Describe the flow of participants through the study, including the number of participants with and without the outcome and, if applicable, a summary of the follow-up time. A diagram may be helpful. | 10, eFigure 1 |
|  | 20b | D;E | Report the characteristics overall and, where applicable, for each data source or setting, including the key dates, key predictors (including demographics), treatments received, sample size, number of outcome events, follow-up time, and amount of missing data. A table may be helpful. Report any  differences across key demographic groups. | 10, Table 1 |
|  | 20c | E | For model evaluation, show a comparison with the development data of the distribution of important predictors (demographics, predictors, and outcome). | N/A |
| *Model development* | 21 | D;E | Specify the number of participants and outcome events in each analysis (e.g., for model development, hyperparameter tuning, model evaluation) | Table 1 |
| *Model specification* | 22 | D | Provide details of the full prediction model (e.g., formula, code, object, application programming interface) to allow predictions in new individuals and to enable third-party evaluation and implementation, including any restrictions to access or re-use (e.g., freely available, proprietary)^5^ | Table 2, Supplementary Methods |
| *Model performance* | 23a | D;E | Report model performance estimates with confidence intervals, including for any key subgroups (e.g., sociodemographic). Consider plots to aid presentation. | Table 3, Figure 1 |
|  | 23b | D;E | If examined, report results of any heterogeneity in model performance across clusters. See TRIPOD  Cluster for additional details^3^. | eTable 7 and 8 |
| *Model updating* | 24 | E | Report the results from any model updating, including the updated model and subsequent performance | N/A |
| **DISCUSSION** | | | | |
| *Interpretation* | 25 | D;E | Give an overall interpretation of the main results, including issues of fairness in the context of the  objectives and previous studies | 13 |
| *Limitations* | 26 | D;E | Discuss any limitations of the study (such as a non-representative sample, sample size, overfitting, missing data) and their effects on any biases, statistical uncertainty, and generalizability | 15 |
| *Usability of the model in the context of current care* | 27a | D | Describe how poor quality or unavailable input data (e.g., predictor values) should be assessed and handled when implementing the prediction model | eTable 2 |
|  | 27b | D | Specify whether users will be required to interact in the handling of the input data or use of the model,  and what level of expertise is required of users | 13-14 |
|  | 27c | D;E | Discuss any next steps for future research, with a specific view to applicability and generalizability of  the model | 14 |

**eTable 2:** Full list of predictors included in the prognostic model with detailed description and specification

| **Predictor** | **Description** | **Modeling categorization** |
| --- | --- | --- |
| Age | Age in years | Age groups of <40, 40-49, 50-59, 60-69, 70-79, 80+ |
| Sex | CDCR categorizes individuals as male, female, and non-binary. The female and non-binary categories were combined into a single category due to the small sample sizes. | Male vs. female |
| Disability Placement Program (DPP) code | CDCR has a Disability Placement Program (DPP), which helps place inmates with disabilities in appropriate housing and programs. Individuals with mobility limitations are assigned into different categories.  The “DPW” category indicates that the individual has severe mobility restrictions and requires a full time wheelchair accommodation to ambulate in and out of cell/bed area.  The “DPO” category indicates that the individual has severe mobility restrictions but only uses a wheelchair intermittently as an accommodation to ambulate outside of cell/bed area.  The “DPM”, “DLT”, and “DNM” categories indicate that the individual has mobility restrictions that do not require wheelchair use to ambulate. For example, DPM indicates that the individual uses an assistive device other than a wheelchair, and DLT indicates that the individual requires a relatively level terrain/path of travel. | Severe mobility restrictions with full-time wheelchair (DPW) vs. severe mobility restrictions with intermittent wheelchair use (DPO) vs. Other mobility restrictions (DPM, DLT, DNM) vs. no DPP code |
| Housing level | Incarcerated individuals are assigned to different housing levels based on several factors, including medical and psychiatric issues. Most individuals are assigned to the general population (GP).  A correctional treatment center (CTC) is a health facility designated to provide health care to individuals who do not require general acute care level of services but are in need of professionally supervised health care beyond that normally provided in the community on an outpatient basis. We term these higher acuity infirmary beds.  An outpatient housing unit (OHU) is a housing unit established to retain inmates who require special housing for security or protection. Typically, these are inmates whose health condition would not normally warrant admission to a licensed heath care facility and for whom housing in the general population may place them at personal or security risk. OHU residents may receive outpatient health services and assistance with the activities of daily living. We term these lower acuity infirmary beds.  Other categories include the restricted housing units and several mental health housing levels defined within the California prison system, including mental health crisis beds, mental health acute care beds, psychiatric inpatient programs, and intermediate care facilities. These were grouped together with the general population housing level as they were not found to be associated with mortality and included relatively few individuals. | General and other housing vs. outpatient housing unit (lower acuity infirmary bed) vs. correctional treatment center (higher acuity infirmary bed) |
| Hospitalizations in the previous year | Number of hospital stays in the 1-year look back period. Hospitalizations occurred outside of CDCR prisons and could be for any cause. | 0, 1, 2+ |
| Any intensive care unit (ICU) admission in the previous year | Any ICU admission during a hospitalization in the 1-year look back period. | Yes vs. No |
| Dialysis | Use of dialysis in the 1-year look back period | Yes vs. No |
| Chronic conditions | CDCR has specific definitions to identify chronic conditions, using a combination of diagnosis codes, medications, and laboratory studies.  For example, individuals with cancer are identified based on an active electronic health record diagnosis related to cancer (based on International Classification of Diseases (ICD) or Systematized Nomenclature of Medicine (SNOMED) codes), a hospitalization or specialty visit claim with a primary diagnosis code related to cancer, or an active prescription for cancer medication.  The total number of available chronic conditions in the dataset was 78.  To reduce the number of comorbidities entered as candidate predictors in the model, we removed any comorbidities with extremely low prevalence (<1%) and contributed to fewer than 10 deaths in our cohort. This resulted in a list of 31 comorbidities which were included as candidate predictors, including: aplastic anemia, asthma, cancer, chronic pain, chronic kidney disease, Coccidioidomycosis, connective tissue disorder, chronic obstructive pulmonary disease, arrhythmia, cerebrovascular disease, congestive heart failure, hypertension, ischemic heart disease, cardiovascular disease-not otherwise specified, peripheral vascular disease, thromboembolic disease, valvular disease, dementia and Parkinson’s disease, diabetes, dyslipidemia, end stage liver disease, glaucoma, hepatitis c, immunosuppressed, osteoporosis, ostomy, pulmonary fibrosis, seizures, sleep apnea, solitary pulmonary nodule, unspecified hepatitis | Yes vs. No to each chronic condition |

**eTable 3:** Classification of the deaths during the 2-year study period from February 1, 2018 to February 1, 2020^*^

| **Death type** | **Number (%)** |
| --- | --- |
| **Natural (expected)** | **372** |
| Autoimmune | 1 |
| Cancer | 213 |
| Cardiovascular disease | 40 |
| Cerebrovascular disease | 3 |
| Circulatory system | 4 |
| Endocrine | 4 |
| Gastrointestinal disease | 2 |
| HIV/AIDS | 2 |
| Infectious disease | 36 |
| Liver disease | 29 |
| Neurological disease | 13 |
| Pulmonary | 19 |
| Renal disease | 6 |
| **Natural (unexpected)** | **121** |
| Adverse medication reaction | 1 |
| Cancer | 10 |
| Cardiovascular disease | 61 |
| Cerebrovascular disease | 4 |
| Circulatory system | 4 |
| Endocrine | 2 |
| Gastrointestinal disease | 5 |
| HIV/AIDS | 1 |
| Infectious disease | 17 |
| Liver disease | 3 |
| Neurological disease | 3 |
| Pulmonary | 8 |
| Renal disease | 2 |
| **Compassionate release (presumed death)^a^** | **13** |
| **Non-natural death** | **204** |
| Accidental injury to self/by other | 6 |
| Drug overdose (accidental) | 101 |
| Drug overdose (suicide) | 2 |
| Homicide | 47 |
| Suicide | 47 |
| Not specified | 1 |

* Our final outcome included 506 deaths (372 natural expected, 121 natural unexpected, and 13 presumed deaths following compassionate release). As the California Department of Corrections and Rehabilitation does not collect information on individuals once they are released from a prison, we did not have information on the date of death for individuals released through the compassionate release program. Therefore, we imputed a death date of 6 months after they were released from the prison based on the experience of CDCR physicians.

**eTable 4:** Univariable association between all predictors and 2-year mortality

| **Characteristic** | **Unadjusted hazard ratio (95% CI)** | **P-value** |
| --- | --- | --- |
| Age group |  |  |
| <40 | 0.01 (0.01, 0.02) | <0.001 |
| 40-49 | 0.08 (0.06, 0.12) | <0.001 |
| 50-59 | 0.33 (0.27, 0.42) | <0.001 |
| 60-69 | Ref. |  |
| 70-79 | 2.26 (1.76, 2.89) | <0.001 |
| 80+ | 6.96 (4.92, 9.85) | <0.001 |
| Sex |  |  |
| Male |  |  |
| Female | 0.69 (0.43, 1.12) | 0.14 |
| Disability Placement Program (DPP) category^*^ |  |  |
| Severe mobility restrictions with full-time wheelchair use | 16.32 (12.34, 21.60) | <0.001 |
| Severe mobility restrictions with intermittent wheelchair use | 16.67 (13.21, 21.05) | <0.001 |
| Other mobility restriction (e.g., assistive device other than wheelchair) | 5.40 (4.44, 6.56) | <0.001 |
| Housing level |  |  |
| General and other housing^†^ | Ref. |  |
| Lower acuity infirmary bed^‡^ | 22.91 (17.23, 30.47) | <0.001 |
| High acuity infirmary bed^‡^ | 60.85 (49.18, 75.30) | <0.001 |
| Hospitalizations in the previous year |  |  |
| 0 | Ref. |  |
| 1 | 4.69 (3.74, 5.88) | <0.001 |
| 2+ | 16.40 (13.36, 20.14) | <0.001 |
| Any ICU admission in the previous year |  |  |
| Yes | 16.26 (9.37, 28.20) | <0.001 |
| No | Ref. |  |
| Dialysis |  |  |
| Yes | 13.27 (6.29, 27.98) | <0.001 |
| No | Ref. |  |
| **Chronic conditions** |  |  |
| Aplastic anemia | 34.16 (18.80, 62.09) | <0.001 |
| Asthma | 1.37 (1.10, 1.72) | 0.006 |
| Cancer | 17.45 (14.52, 20.97) | <0.001 |
| Chronic pain | 4.02 (3.37, 4.80) | <0.001 |
| Chronic kidney disease | 6.51 (5.45, 7.78) | <0.001 |
| Coccidioidomycosis | 1.25 (0.75, 2.10) | 0.39 |
| Connective tissue disorder | 3.33 (1.88, 5.91) | <0.001 |
| Chronic obstructive pulmonary disease | 10.28 (8.49, 12.45) | <0.001 |
| Arrhythmia | 10.50 (8.47, 13.01) | <0.001 |
| Cerebrovascular disease | 9.01 (6.73, 12.06) | <0.001 |
| Congestive heart failure | 16.79 (12.98, 21.71) | <0.001 |
| Hypertension | 5.49 (4.57, 6.61) | <0.001 |
| Ischemic heart disease | 9.76 (7.94, 11.98) | <0.001 |
| Cardiovascular disease not otherwise specified | 2.25 (1.48, 3.42) | <0.001 |
| Peripheral vascular disease | 11.61 (8.92, 15.12) | <0.001 |
| Thromboembolic disease | 11.32 (8.23, 15.58) | <0.001 |
| Valvular disease | 7.57 (4.84, 11.84) | <0.001 |
| Dementia & Parkinson’s disease | 15.10 (10.81, 21.10) | <0.001 |
| Diabetes | 5.35 (4.45, 6.44) | <0.001 |
| Dyslipidemia | 4.25 (3.56, 5.07) | <0.001 |
| End stage liver disease | 15.21 (12.33, 18.77) | <0.001 |
| Glaucoma | 3.13 (2.39, 4.10) | <0.001 |
| Hepatitis C | 2.23 (1.86, 2.67) | <0.001 |
| Immunosuppressed | 7.98 (5.94, 10.72) | <0.001 |
| Osteoporosis | 10.70 (6.77, 16.92) | <0.001 |
| Ostomy | 12.79 (7.38, 22.19) | <0.001 |
| Pulmonary fibrosis | 22.18 (11.86, 41.47) | <0.001 |
| Seizures | 2.35 (1.79, 3.08) | <0.001 |
| Sleep apnea | 3.10 (2.25, 4.27) | <0.001 |
| Solitary pulmonary nodule | 6.47 (4.14, 10.13) | <0.001 |
| Unspecified hepatitis | 3.44 (2.20, 5.38) | <0.001 |

Abbreviations: CI, confidence interval; ICU, intensive care unit

* The disability placement program classifies individuals based on mobility restrictions. See eTable 2 in the Supplement for additional details.

† This category includes individuals in restricted housing units and mental health housing levels

‡ In the California Department of Corrections and Rehabilitation, higher acuity infirmary beds are termed correctional treatment centers (CTC), providing care to individuals who need professionally supervised health care. Lower acuity infirmary beds are termed outpatient housing units (OHU), providing care to individuals who may need some assistance which places them at personal or security risk in the general population.

**eTable 5.** Comparison in model performance among the modeling approaches in predicting 2-year mortality for incarcerated individuals^*^

|  |  | 2-year AUC (95% CI) | Intercept  (95% CI) | Slope (95% CI) | Integrated calibration index (Eavg) (95% CI) |
| --- | --- | --- | --- | --- | --- |
| Cox proportional hazards regression | Full model | 0.924 (0.914, 0.933) | 0.0800 (0.0798, 0.08025) | 0.953 (0.953, 0.953) | 0.0015 (0.0010, 0.0019) |
|  | Constrained LASSO^†^ | 0.926 (0.915, 0.938) | 0.0690 (0.0689, 0.0692) | 0.968 (0.967, 0.968) | 0.0014 (0.0010, 0.0018) |
| Logistic regression without accounting for loss to follow-up | Full model | 0.930 (0.919, 0.941) | -0.087 (-0.087, -0.087) | 0.973 (0.973, 0.973) | 0.0012 (0.0008, 0.0015) |
|  | Constrained LASSO | 0.932 (0.922, 0.942) | -0.048 (-0.048, -0.048) | 0.980 (0.980, 0.980) | 0.0012 (0.0008, 0.0016) |
| Logistic regression with exposure offset time based on the first time of release date only | Full model | 0.874 (0.857, 0.891) | -0.961 ( -1.084, -0.837) | 0.747 (0.721, 0.774) | 0.0022 (0.0016, 0.0028) |
|  | Constrained LASSO | 0.873 (0.856, 0.891) | -0.959 (-1.059, -0.859) | 0.747 (0.724, 0.769) | 0.0022 (0.0016, 0.0027) |
| Logistic regression with exposure offset time using all available follow-up time | Full model | 0.872 (0.855, 0.890) | -1.004 (-1.104, -0.904) | 0.739 (0.717, 0.760) | 0.0023 (0.0017, 0.0028) |
|  | Constrained LASSO | 0.874 (0.853, 0.895) | -0.935 (-1.034, -0.836) | 0.750 (0.728, 0.772) | 0.0022 (0.0017, 0.0027) |

Abbreviations: AUC, area under the receiver operating characteristic curve; CI, confidence interval; LASSO, Least Absolute Shrinkage and Selection Operator

* All reported measures have been optimism-corrected through bootstrapping.

† This model represents the final model for the primary analysis (constrained LASSO-Cox model).

**eTable 6.** Performance metrics at different 2-year mortality risk thresholds for the modeling approaches

| Modeling approach | 2-year mortality risk threshold | Total individuals above threshold | Deaths identified (out of 506 deaths) | Positive predictive value | Negative predictive value | Sensitivity | Specificity |
| --- | --- | --- | --- | --- | --- | --- | --- |
| Cox proportional hazards regression | Full model |  |  |  |  |  |  |
|  | >5% | 1552 | 245 | 17.0% | 99.7% | 47.8% | 98.5% |
|  | >10% | 785 | 170 | 23.3% | 99.6% | 33.0% | 99.3% |
|  | >20% | 348 | 103 | 30.8% | 99.5% | 19.9% | 99.7% |
|  | >60% | 62 | 32 | 54.6% | 99.4% | 6.0% | 99.9% |
|  | Constrained LASSO |  |  |  |  |  |  |
|  | >5% | 1558 | 244 | 16.7% | 99.7% | 47.6% | 98.4% |
|  | >10% | 773 | 168 | 23.4% | 99.6% | 32.5% | 99.3% |
|  | >20% | 355 | 103 | 30.2% | 99.5% | 19.9% | 99.7% |
|  | >60% | 62 | 30 | 50.6% | 99.4% | 5.6% | 99.9% |
| Logistic regression without accounting for loss to follow-up | Full model |  |  |  |  |  |  |
|  | >5% | 1523 | 244 | 16.0% | 99.7% | 48.2% | 98.6% |
|  | >10% | 788 | 176 | 22.3% | 99.6% | 34.8% | 99.3% |
|  | >20% | 364 | 109 | 29.9% | 99.6% | 21.5% | 99.7% |
|  | >60% | 46 | 22 | 47.8% | 99.5% | 4.3% | 99.9% |
|  | Constrained LASSO |  |  |  |  |  |  |
|  | >5% | 1509 | 244 | 16.2% | 99.7% | 48.2% | 98.6% |
|  | >10% | 779 | 173 | 22.2% | 99.6% | 34.2% | 99.3% |
|  | >20% | 359 | 104 | 29.0% | 99.5% | 20.6% | 99.7% |
|  | >60% | 49 | 23 | 46.9% | 99.46% | 4.5% | 99.9% |
| Logistic regression with exposure offset time based on the first time of release date only | Full model |  |  |  |  |  |  |
|  | >5% | 1552 | 169 | 10.9% | 99.6% | 33.4% | 98.4% |
|  | >10% | 789 | 120 | 15.2% | 99.6% | 23.7% | 99.2% |
|  | >20% | 393 | 70 | 17.8% | 99.5% | 13.8% | 99.6% |
|  | >60% | 51 | 12 | 23.5% | 99.4% | 2.4% | 99.9% |
|  | Constrained LASSO |  |  |  |  |  |  |
|  | >5% | 1567 | 169 | 10.8% | 99.6% | 33.4% | 98.4% |
|  | >10% | 802 | 114 | 14.2% | 99.6% | 22.5% | 99.2% |
|  | >20% | 385 | 70 | 18.2% | 99.5% | 13.8% | 99.6% |
|  | >60% | 48 | 10 | 20.8% | 99.4% | 2.0% | 99.9% |
| Logistic regression with exposure offset time using all available follow-up time | Full model |  |  |  |  |  |  |
|  | >5% | 1551 | 169 | 10.9% | 99.6% | 33.4% | 98.4% |
|  | >10% | 789 | 120 | 15.2% | 99.6% | 23.7% | 99.2% |
|  | >20% | 393 | 70 | 17.8% | 99.5% | 13.8% | 99.6% |
|  | >60% | 51 | 12 | 23.5% | 99.4% | 2.4% | 99.9% |
|  | Constrained LASSO |  |  |  |  |  |  |
|  | >5% | 1567 | 169 | 10.8% | 99.6% | 33.4% | 98.4% |
|  | >10% | 802 | 114 | 14.2% | 99.6% | 22.5% | 99.2% |
|  | >20% | 385 | 70 | 18.2% | 99.5% | 13.8% | 99.6% |
|  | >60% | 48 | 10 | 20.8% | 99.4% | 2.0% | 99.9% |

Abbreviations: LASSO, Least Absolute Shrinkage and Selection Operator

**eTable 7:** Model performance and fairness evaluation for a LASSO Cox model with and without race and ethnicity at a threshold of 5% 2-year mortality risk

|  | N (%) | 2-year AUC (95% CI) | Integrated calibration index (ICI) (95% CI) | Sensitivity (true positive rate)*  (95% CI) | Equal opportunity difference^†^ (95% CI) | Positive predictive value^‡^ (95% CI) | Predictive parity difference^§^  (95% CI) | Disparate impact^\|\|^  (95% CI) |
| --- | --- | --- | --- | --- | --- | --- | --- | --- |
| Final model without race and ethnicity as a predictor | 89,430 | 0.926 (0.915, 0.938) | 0.0014 (0.0010, 0.0018) | 47.6% (42.3%, 51.6%) | NA | 16.7% (14.3%, 18.5%) | NA | NA |
| Non-Hispanic Black | 27,383 (30.6%) | 0.910 (0.886, 0.933) | 0.0006 (-0.0001, 0.0013) | 49.0% (38.4%, 59.6%) | -2.1% (-3.9,  -0.2%) | 16.5% (11.8%, 21.2%) | 1.2% (0.5%, 2.0%) | 0.96 (0.92, 0.99) |
| Hispanic | 37,047 (41.4%) | 0.930 (0.909, 0.952) | 0.0007 (0.0001, 0.0012) | 40.1% (31.6%, 48.7%) | -10.9% (-12.5%, -9.3%) | 18.5% (14.3%, 22.6%) | 3.3% (2.6%, 4.0%) | 0.79 (0.76, 0.82) |
| Non-Hispanic White | 18,497 (20.7%) | 0.907 (0.889, 0.925) | 0.0030 (0.0019, 0.0042) | 51.1% (43.4%, 58.7%) | 0 | 15.2% (12.7%, 17.8%) | 0 | 1 |
| Other^¶^ | 6,503 (7.27%) | 0.945 (0.915, 0.974) | 0.0018 (-0.0004, 0.0039) | 45.1% (29.8%, 60.4%) | -5.9% (-8.4%,  -3.5%) | 20.4% (11.8%, 29.1%) | 5.2% (3.9%, 6.5%) | 0.88 (0.84, 0.93) |
| Model with race and ethnicity as a predictor | 89,430 | 0.925 (0.913, 0.938) | 0.0014 (0.0010, 0.0018) | 47.3% (43.5%, 51.0%) | NA | 16.8% (14.8%, 18.1%) | NA | NA |
| Non-Hispanic Black | 27,383 (30.6%) | 0.905 (0.883, 0.927) | 0.0005 (-0.0004, 0.0014) | 45.9% (35.6%, 56.2%) | -1.6%(-3.6%,  -0.3%) | 18.6% (15.4%, 21.9%) | 3.5% (2.9%, 4.1%) | 0.97 (0.93, 1.01) |
| Hispanic | 37,047 (41.4%) | 0.930 (0.908, 0.953) | 0.0006 (-0.0001, 0.0012) | 43.0% (33.8%, 52.1%) | -4.6%(-6.4%,  -2.7%) | 16.5% (13.0%, 20.0%) | 1.4% (0.8%, 2.0%) | 0.90 (0.87, 0.94) |
| Non-Hispanic White | 18,497 (20.7%) | 0.909 (0.888, 0.930) | 0.0033 (0.0021, 0.0045) | 47.5% (38.3%, 56.8%) | 0 | 15.1% (12.3%, 18.0%) | 0 | 1 |
| Other^f^ | 6,503 (7.27%) | 0.920 (0.888, 0.951) | -0.0007 (-0.0029, 0.0015) | 36.5% (20.7%, 52.4%) | -11.0% (-13.6%, -8.4%) | 17.4% (12.6%, 22.2%) | 2.3% (1.5%, 3.1%) | 0.77 (0.72, 0.82) |

Abbreviations: AUC, area under the receiver operating characteristic curve; CI, confidence interval

* Sensitivity, also known as the true positive rate, refers to the proportion of individuals with a condition who are correctly identified as positive. In the context of this study, it refers to the proportion of individuals who died at 2 years who were correctly predicted as high risk (2-year mortality >5%).

† Equal opportunity difference is defined as the difference in true positive rates between non-White groups (Black, Hispanic, Other) and White groups. The target value for equal opportunity difference is 0.

‡ Positive predictive value, also known as precision, refers to the proportion of individuals with a positive test result that are actually true positives. In the context of this study, it refers to the proportion of individuals who were predicted as high risk (2-year mortality >5%) who died at 2 years.

§ Predictive parity difference is defined as the difference in positive predictive values between non-White groups (Black, Hispanic, Other) and White groups. The target value for predictive parity difference is 0.

|| Disparate impact is a measure of statistical parity that assesses the ratio of the proportions of positive predictions for the non-White group (American Indian, Black, Hispanic) to that of the White group. In other words, based on disparate impact, the ratio of the proportions of individuals classified as high risk (2-year mortality >5%) in non-White and White groups would have a target value of 1.

¶ Other includes American Indian/Alaskan Native, Asian/Pacific Islander, and Other/unknown

**eTable 8:** Model performance and fairness evaluation for a LASSO Cox model within subgroups by race and ethnicity and sex at threshold of 5% 2-year mortality risk

|  | N (%) | Number of deaths (% within subgroup) | 2-year AUC (95% CI) | Integrated calibration index (95% CI) | Sensitivity (true positive rate)* (95% CI) | Specificity (95% CI) | Positive predictive value^†^ (95% CI) | Negative predictive value (95% CI) |
| --- | --- | --- | --- | --- | --- | --- | --- | --- |
| Full cohort | 89,430 | 506 (0.6%) | 0.926 (0.915, 0.938) | 0.0014 (0.0010, 0.0018) | 47.6%  (42.3%, 51.6%) | 98.4%  (98.2%, 98.4%) | 16.7%  (14.3%, 18.5%) | 99.7%  (99.6%, 99.7%) |
| Race and ethnicity |  |  |  |  |  |  |  |  |
| Non-Hispanic Black | 27,383 (30.6%) | 127 (0.5%) | 0.910 (0.886, 0.933) | 0.0006 (-0.0001, 0.0013) | 49.0% (38.4%, 59.6%) | 98.7% (98.4%, 98.9%) | 16.5% (11.8%, 21.2%) | 99.7% (99.7%, 99.8%) |
| Hispanic | 37,047 (41.4%) | 131 (0.4%) | 0.930 (0.909, 0.952) | 0.0007 (0.0001, 0.0012) | 40.1% (31.6%, 48.7%) | 99.3% (99.1%, 99.4%) | 18.5% (14.3%, 22.6%) | 99.8% (99.7%, 99.8%) |
| Non-Hispanic White | 18,497 (20.7%) | 203 (1.1%) | 0.907 (0.889, 0.925) | 0.0030 (0.0019, 0.0042) | 51.1% (43.4%, 58.7%) | 96.1% (95.6%, 96.7%) | 15.2% (12.7%, 17.8%) | 99.3% (99.2%, 99.4%) |
| Other^‡^ | 6,503 (7.27%) | 45 (0.7%) | 0.945 (0.915, 0.974) | 0.0018 (-0.0004, 0.0039) | 45.1% (29.8%, 60.4%) | 98.6% (98.2%, 99.0%) | 20.4% (11.8%, 29.1%) | 99.6% (99.4%, 99.7%) |
| Sex |  |  |  |  |  |  |  |  |
| Male | 84,999 (95.0%) | 489 (0.6%) | 0.927 (0.917, 0.938) | 0.0014 (0.0010, 0.0019) | 46.9% (42.6%, 51.2%) | 98.4% (98.2%, 98.6%) | 16.4% (14.5%, 18.4%) | 99.6% (99.6%, 99.7%) |
| Female | 4,431 (5.0%) | 17 (0.4%) | 0.916 (0.857, 0.975) | -0.0005 (-0.0031, 0.0021) | 39.0% (8.7%, 69.3%) | 99.1% (98.3%, 99.9%) | 16.7% (5.4%, 28.1%) | 99.7% (99.6%, 99.9%) |

Abbreviations: AUC, area under the receiver operating characteristic curve; CI, confidence interval

* Sensitivity refers to the proportion of individuals who died at 2-years who were flagged as having a 2-year mortality risk >5%. The equal opportunity fairness metric evaluates whether sensitivity (true positive rate) is similar across subgroups. Equal opportunity is defined as when, within each subgroup, there is an equal probability of an individual in a positive class (dies within 2 years) to have a positive prediction (2-year mortality risk >5%). This prioritizes equal error rates across groups.

† Positive predictive value refers to the proportion of individuals who were flagged as having a 2-year mortality risk >5% that had a natural death at 2 years. The predictive parity fairness metric evaluates whether the positive predictive value is similar across subgroups. Predictive parity is defined as when, within each subgroup, there is an equal probability of an individual with a positive prediction (2-year mortality risk >5%) to truly belong to the positive class (dies within 2 years). This prioritizes equal accuracy of positive predictions across groups.

‡ Other includes American Indian/Alaskan Native, Asian/Pacific Islander, and Other/unknown

**eTable 9**: Baseline characteristics for 10 randomly selected individuals based on predicted 2-year mortality risk

| **Individual** | **1** | **2** | **3** | **4** | **5** | **6** | **7** | **8** | **9** | **10** |
| --- | --- | --- | --- | --- | --- | --- | --- | --- | --- | --- |
| **Characteristic** |  |  |  |  |  |  |  |  |  |  |
| Age group | 50-59 | 40-49 | 50-59 | 50-59 | 80+ | 70-79 | 60-69 | 60-69 | 60-69 | 70-79 |
| Sex | Male | Male | Male | Male | Male | Male | Male | Male | Male | Male |
| Disability Placement Program category | None | Severe mobility restriction with full-time wheelchair | Severe mobility restriction with full-time wheelchair | Severe mobility with intermittent wheelchair | Severe mobility restriction with full-time wheelchair | Severe mobility restriction with full-time wheelchair | Severe mobility restriction with full-time wheelchair | Other mobility restriction | Other mobility restriction | Severe mobility with intermittent wheelchair |
| Housing level | General housing | Higher acuity infirmary bed | Higher acuity infirmary bed | General housing | Higher acuity infirmary bed | Higher acuity infirmary bed | Higher acuity infirmary bed | General housing | Lower acuity infirmary bed | Higher acuity infirmary bed |
| Hospitalizations in past year | 0 | 1 | 0 | 2+ | 0 | 2+ | 2+ | 2+ | 2+ | 1 |
| Intensive care unit stay in past year | 0 | 0 | 0 | 0 | 0 | 0 | 0 | 0 | 0 | 0 |
| Dialysis | 0 | 0 | 0 | 0 | 0 | 0 | 0 | 0 | 0 | 0 |
| Chronic conditions (listed) | ESLD, HCV | COPD | Chronic pain, Ostomies | Chronic pain, CKD, COPD, ESLD, HCV | CKD, PVD, Diabetes | CKD, Arrhythmia | Chronic pain, CKD, COPD, Diabetes, Ostomies | Cancer, Chronic pain, CKD, COPD, ESLD, HCV, Immunosuppressed | Cancer, CKD, Arrhythmia, Thromboembolic disease, Diabetes, Ostomies | Cancer, Chronic pain, CKD, Diabetes, HCV |
| **2-year mortality risk** | **1%** | **2%** | **5%** | **10%** | **15%** | **20%** | **30%** | **40%** | **50%** | **60%** |

Abbreviations: CKD, chronic kidney disease; COPD, chronic obstructive pulmonary disease; ESLD, end stage liver disease; HCV, hepatitis C virus; PVD, peripheral vascular disease
